# Ultra-rapid peritoneal dissemination of a somatic STK11-mutant adnexal tumor: a case report with molecular correlation and literature review

**DOI:** 10.3389/fonc.2026.1836973

**Published:** 2026-07-24

**Authors:** Yaxin Wang, Jiongbo Liao, Yiqin Wang, Mengyu Chu, Fenghua Ma, Jia Liu, Xiang Tao, Chao Wang

**Affiliations:** 1 Department of Obstetrics & Gynecology, Obstetrics & Gynecology Hospital of Fudan University, Shanghai, China; 2 Obstetrics & Gynecology Hospital of Fudan University, Shanghai Key Lab of Reproduction and Development, Shanghai, China; 3 Obstetrics & Gynecology Hospital of Fudan University, Shanghai Key Lab of Female Reproductive Endocrine Related Diseases, Shanghai, China; 4 Department of Pathology, Obstetrics & Gynecology Hospital of Fudan University, Shanghai, China; 5 Department of Radiology, Obstetrics & Gynecology Hospital of Fudan University, Shanghai, China

**Keywords:** case report, molecular pathology, peritoneal carcinomatosis, Peutz-Jeghers syndrome, STK11 adnexal tumor

## Abstract

STK11-adnexal tumor (STK11-AT) is a recently characterized rare neoplasm tightly linked to Peutz-Jeghers syndrome (PJS) and marked by high recurrence potential. We report a 38-year-old woman who presented with a pelvic mass 9 months after resection of a primary left fallopian tube tumor. Initial pathology suggested a malignant Wolffian duct tumor, but expert pathological review and molecular profiling confirmed STK11-AT harboring a somatic STK11 nonsense mutation (c.367C>T, p.Gln123*), identified via next-generation sequencing (NGS) with a variant allele frequency (VAF) of 88.55%. Despite postoperative normalization of serum CA-125, surveillance imaging revealed ultra-rapid recurrence with extensive peritoneal carcinomatosis and retroperitoneal lymph node metastasis, necessitating comprehensive cytoreductive surgery. Recurrent lesions exhibited a high Ki-67 proliferation index (70%), consistent with aggressive biological behavior. This case expands the clinicopathological spectrum of STK11-AT by documenting hyperacute progression (shorter than the reported median recurrence interval of 17 months) and highlights the critical role of definitive molecular testing for diagnosis, as well as the need for intensive long-term surveillance even after R0 resection.

## 1 Introduction

STK11 (serine/threonine kinase 11), also known as LKB1, encodes a tumor suppressor protein that regulates cell metabolism, proliferation, and polarity via activation of downstream pathways such as AMP-activated protein kinase (AMPK) (1). Germline STK11 mutations cause Peutz-Jeghers syndrome (PJS), an autosomal dominant disorder characterized by gastrointestinal hamartomatous polyposis and elevated risk of multiple cancers (2). STK11-adnexal tumor (STK11-AT)—first delineated as a distinct entity by Bennett et al. in 2021 (2)—is a rare neoplasm of the female genital tract defined by STK11 alterations (somatic or germline) and a unique morphologic/immunophenotypic profile.

Clinically, STK11-AT often mimics female adnexal tumors of Wolffian origin (FATWO) or ovarian sex cord-stromal tumors, posing significant diagnostic challenges (3). A landmark study of 22 STK11-AT cases reported an 80% recurrence rate, with peritoneal dissemination as the most common pattern and a median time to recurrence of 17 months (2). Notably, ~45% of STK11-AT patients have concurrent PJS or germline STK11 mutations, and these PJS-associated tumors tend to be larger (mean 12.6 cm vs. 8.9 cm) and have longer recurrence intervals (median 19 months vs. 7 months) than non-PJS counterparts (4).

Given the limited clinical data on non-PJS, somatic STK11-mutant STK11-AT and its heterogeneous aggressiveness, we present a case of ultra-rapid peritoneal dissemination (9 months post-R0 resection) with a high Ki-67 index. This report aims to refine understanding of STK11-AT’s biological behavior, diagnostic criteria, and surveillance strategies—critical unmet needs for managing this aggressive neoplasm.

## 2 Case presentation

**Figure 1:**
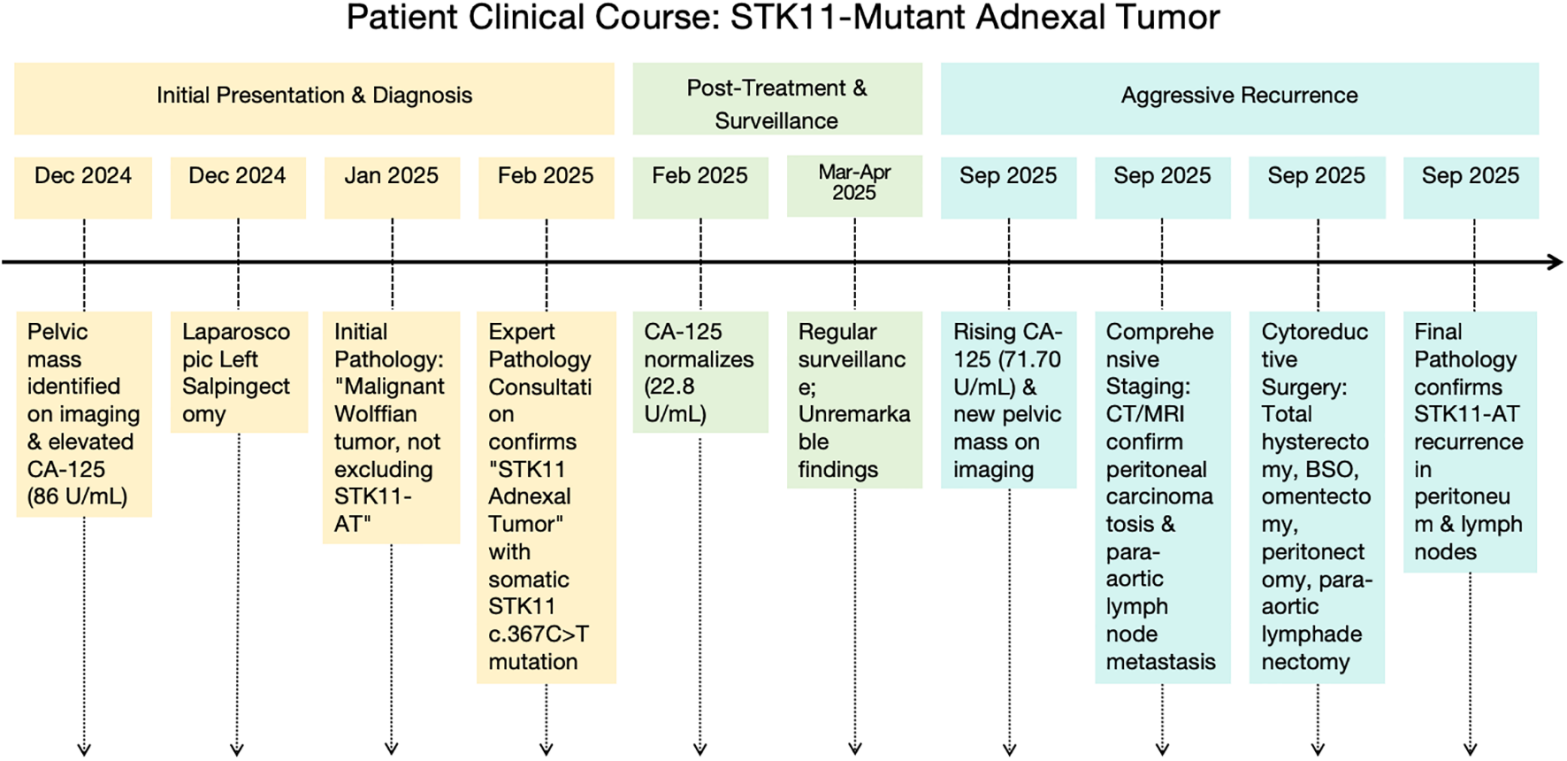
Timeline flowchart: clinical course of a patient with STK11-mutant adnexal tumor. STK11-AT, STK11 Adnexal Tumor; BSO, Bilateral Salpingo-Oophorectomy (removal of both fallopian tubes and ovaries); CT, Computed Tomography; MRI, Magnetic Resonance Imaging; TC, Albumin paclitaxel combined with carboplatin chemotherapy regimen; ICI, Tislelizumab.

A 38-year-old woman (gravida 2, para 2) was admitted to our institution on September 4, 2025, for evaluation of a newly identified pelvic mass. Her clinical course is summarized in **Figure 1**. This flowchart visualizes the diagnostic and therapeutic journey of a 38-year-old female with a rapidly progressing STK11-mutant adnexal tumor.

### 2.1 Initial diagnosis and primary surgery (May 2024–December 2024)

In May 2024 (42 days postpartum), a 4 cm pelvic mass was incidentally detected on routine postpartum ultrasound and monitored serially. By December 2024, follow-up ultrasound showed an enlarged left adnexal complex mass (63×54×46 mm), and pelvic MRI confirmed a left parauterine cystic-solid lesion with peripheral enhancement. Serum CA-125 was elevated to 86 U/mL (normal range: 0–35 U/mL), while other tumor markers (CEA, CA19-9, AFP) were within normal limits.

On December 31, 2024, the patient underwent laparoscopic left salpingectomy at Jiangxi Maternal and Child Health Hospital. Intraoperatively, a 6×5 cm solid-cystic tumor was isolated in the left fallopian tube and extracted in a retrieval bag to prevent peritoneal seeding. Initial pathology suggested “malignant Wolffian duct tumor, with STK11-AT not excluded,” prompting referral to Fudan University Shanghai Cancer Center for second-opinion review.

### 2.2 Molecular confirmation and germline testing (February 2025–April 2025)

Postoperatively, CA-125 normalized to 22.8 U/mL (February 4, 2025). The patient sought a second opinion at Fudan University Shanghai Cancer Center. Pathology review and molecular testing confirmed “(Left fallopian tube) adnexal tumor with STK11 gene mutation.” Molecular analysis identified a somatic STK11 nonsense mutation in exon 2 (c.367C>T, p.Gln123*), with a variant allele frequency of 88.55%. Subsequently, peripheral blood germline gene testing performed in our hospital in April 2025 reported that “no pathogenic mutations were detected in 35 genes (including STK11)”, which ruled out PJS.

### 2.3 Surveillance and recurrence detection (March 2025–September 2025)

Surveillance was initiated with serial imaging and tumor marker monitoring. A pelvic ultrasound on March 8, 2025, was unremarkable. However, on September 2, 2025, follow-up ultrasound identified a 59×45×33 mm mass anterior to the uterus, and the patient was admitted to our hospital for further evaluation.

### 2.4 Diagnostic assessments on readmission (September 2025)

Tumor markers: Serum CA-125 was re-elevated to 71.70 U/mL; CEA, CA19-9, and AFP remained normal.

Imaging: Chest CT showed multiple pulmonary air cysts (consistent with benign emphysematous changes) but no metastatic nodules. Abdominal/pelvic contrast-enhanced CT revealed multiple retroperitoneal nodules (largest: 3.0×1.9 cm) with homogeneous enhancement, highly suggestive of metastasis. Pelvic MRI (T2-weighted imaging) confirmed a 5.49×3.29 cm hyperintense mass inferior to the uterus, with peripheral enhancement on post-contrast sequences—consistent with recurrent STK11-AT.

### 2.5 Surgical intervention and intraoperative findings (September 5, 2025)

The patient underwent exploratory laparotomy with comprehensive cytoreductive surgery, including:

total abdominal hysterectomy, bilateral salpingo-oophorectomy, peritoneal lesion resection (bladder peritoneum, pelvic sidewalls, pouch of Douglas, diaphragm, paracolic gutters), infracolic omentectomy Para-aortic lymphadenectomy (up to the renal vessels).

Intraoperative findings included: widespread miliary nodules (1–3 mm) on the diaphragmatic surface, pelvic peritoneum, and paracolic gutters (peritoneal carcinomatosis), a 5.5×4×4 cm firm, well-circumscribed mass in the right vesicouterine pouch. There were 11 enlarged and hard para-aortic lymph nodes at the level of renal vessels, with a diameter of 0.5-3.0 cm.

Intraoperative frozen section analysis of omental and peritoneal nodules confirmed malignant infiltration. Complete cytoreduction was achieved, with no gross residual disease (R0 resection).

### 2.6 Pathological and immunohistochemical findings (September 15, 2025)

**Figure 2:**
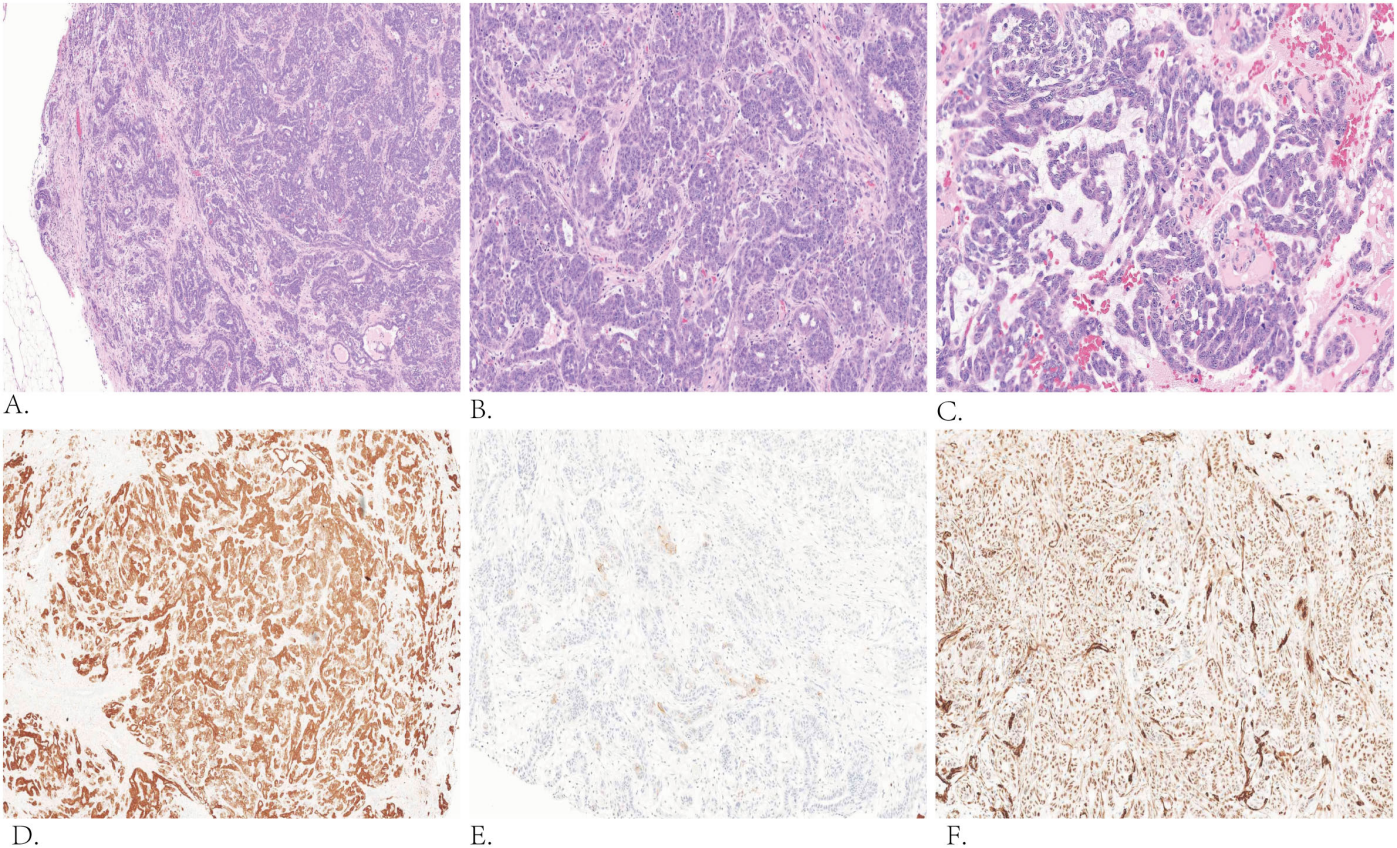
Morphological and immunohistochemical analysis of the metastatic omental tumor from this patient. **(A–C)** round cells arranged in tubular structures (HE staining), **(A–C)** were 4, 10 and 20 times larger than under the microscope, respectively. **(D)** Calretinin(+); **(E)** CD10(Focal +); **(F)** WT-1(+). The red circle is shown High-grade nuclear atypia.

Final pathology confirmed STK11-AT involvement of the bladder peritoneum, pelvic sidewall peritoneum, omentum, pouch of Douglas, and diaphragmatic surface. Four of 11 resected para-aortic lymph nodes contained metastatic STK11-AT. The specific IHC results of the pathological specimen from this patient are shown in **Table 1**. The specific morphology of the tumor cells in this patient under different staining conditions is shown in **Figure 2**. The cells were often arranged in a variety of morphologies, as shown in **Figure 2C**, with clearly anastomostic cell cords and trabeculae, and they could also form tubular, cystic, cribriform, or microacinoid structures.

**Table 1:** A pattern pathognomonic for STK11-AT.

Marker	Staining pattern	Positivity	Clinical significance
ER	Nuclear	80%	Consistent with STK11-AT phenotype (2)
PR	Nuclear	Negative	Typical of STK11-AT (2)
WT-1	Nuclear	Positive	Differentiates from mesothelioma (3)
Calretinin	Cytoplasmic	Positive	Key marker for STK11-AT (2)
Inhibin-α	Cytoplasmic	Negative	Differentiates from sex cord tumors
PAX-8	Nuclear	Negative	Differentiates from FATWO (3)
CD10	Cytoplasmic	Focal	Variable in STK11-AT (4)
Ki-67	Nuclear	70%	Indicates high proliferation (1)
p53	Nuclear	Wild-type	No additional TP53 mutation
MLH1/MSH2/MSH6/PMS2	Nuclear	Intact	No mismatch repair deficiency

NGS re-testing of recurrent tumor tissue confirmed the identical somatic STK11 c.367C>T mutation (VAF = 85.2%), confirming clonal recurrence. Peritoneal washing cytology was positive for malignant cells (consistent with adenocarcinoma morphology).

### 2.7 Postoperative chemotherapy and follow-up (September 29, 2025–March 2026)

The patient had a good postoperative recovery, and the first chemotherapy was started on September 29, 2025. After excluding the contraindication of chemotherapy, TC+ICI regimen: albumin paclitaxel + carboplatin + tislelizumab (anti-PD-1) combined therapy, supplemented by liver protection and stomach protection and antiemetic therapy. A total of 6 cycles of combination chemotherapy were completed. Tislelizumab monotherapy was initiated as maintenance therapy in March 2026. The patient is currently closely followed up for the results of imaging and tumor markers, and there are no significant abnormalities.

## 3 Discussion

The STK11 gene is located on chromosome 19p13.3, which is mainly responsible for the phosphorylation of at least 14 proteins and plays a crucial role in tumor suppression (5). Inactivation or mutation of STK11 usually results in an autosomal dominant disorder called Peutz-Jeghers syndrome (PJS), which has been reported to occur in approximately 1 in 50,000 to 1 in 250,000 live-born infants (6). This case advances understanding of STK11-AT by highlighting three novel clinical and mechanistic insights: (1) ultra-rapid recurrence (9 months post-R0 resection) in a somatic STK11-mutant, non-PJS patient; (2) a high Ki-67 index (70%) as a potential prognostic marker for aggressive disease; and (3) familial cancer clustering in a somatic STK11-mutant patient, challenging conventional models of heredity in STK11-AT.

### 3.1 Ultra-rapid progression: evidence for phenotypic heterogeneity

The 9-month recurrence interval in our patient is substantially shorter than the median 17 months reported by Bennett et al. (2) and the 7-month median for non-PJS STK11-AT reported by Wu et al. (4). This hyperacute progression supports the existence of an “aggressive subtype” of STK11-AT, distinct from the more indolent variants described in prior studies.

The extremely high Ki-67 index (70%) in recurrent lesions likely drives this aggressiveness. STK11/LKB1 normally suppresses cell proliferation by activating AMPK, which inhibits mTOR signaling and cell cycle progression (1). Loss of STK11 function (via the c.367C>T nonsense mutation, which truncates the LKB1 protein) disrupts this pathway, leading to unchecked cell division—consistent with the 70% Ki-67 index. This suggests that Ki-67 may play a role as a prognostic biomarker for STK11-AT. For example, according to a certain threshold (e.g., ≥50%), patients who may be at risk of ultra-rapid recurrence can be identified in advance, and then the frequency of surveillance for these patients can be increased. Of course, this case is limited by a single case report and does not have the ability to determine the threshold, and more cases from all over the world need to be summarized for further discussion.

### 3.2 Diagnostic challenges: the critical role of molecular testing

The morphology of STK11 adjoint tumors overlaps with FATWO, but STK11 adjoint tumors are more aggressive and have a poor prognosis, while FATWO usually has a benign clinical course. The differential diagnosis of STK11 adnexal tumors from sex cord-stromal tumors and mesothelioma depends more on molecular detection. STK11-AT’s morphologic and immunophenotypic overlap with FATWO, sex cord-stromal tumors, and peritoneal mesothelioma makes definitive diagnosis difficult without molecular testing (3). Keyhanian et al. (3) reported that 14% of STK11-AT/FATWO cases are initially misdiagnosed as mesothelioma, due to overlapping expression of calretinin and WT-1. In our case, initial pathology suggested a malignant Wolffian duct tumor, but immunostaining (calretinin+/inhibin-α-/WT-1+) and STK11 mutation testing confirmed STK11-AT.

This underscores a critical clinical recommendation: all adnexal tumors with ambiguous morphology (especially those resembling FATWO or mesothelioma) should undergo STK11 mutation testing (via NGS or Sanger sequencing) to rule out STK11-AT. Immunohistochemistry alone is insufficient, as demonstrated by the overlapping markers reported in prior studies (3). With the development of technology, next-generation sequencing (NGS) has been found to be helpful in identifying genetic alterations associated with ovarian cancer, including mutations in TP53, PTEN, and STK11 (7). For example, the risk of OC in PJS patients with STK11 mutations is approximately 18 to 21%(39 to 44% at 70 years of age) (10). Previously, BRCA1/2 was the best known gene originally identified to be associated with hereditary OC (8, 9). This has greatly enriched our understanding of the association between ovarian cancer and various genetic disorders.

### 3.3 Familial cancer history: rethinking “sporadic” STK11-AT

The germline mutation rate of STK11 is usually high in PJS patients, and most studies have established the association of STK11 with lung, pancreatic, gastric, and colorectal tumors (10–12). Gynecological manifestations are often sex cord tumors with annular tubules (sctat) in the ovary and lobular endocervical adenoid hyperplasia (LEGH)/minimal deviation adenocarcinoma (MDA) in the cervix (13, 14). Our patient had a significant family history of cancer (paternal liver cancer, maternal grandmother’s colorectal cancer, maternal aunt’s gastric cancer) despite negative germline STK11 testing. This challenges the classic dichotomy of “germline mutation = hereditary” vs. “somatic mutation = sporadic” in STK11-AT.

The 57-gene germline panel used in this study was limited in scope; a comprehensive panel (e.g., 500+ cancer-related genes) could identify variants in genes that interact with STK11/LKB1 (e.g., PTEN, TP53) or independently increase cancer risk. Such testing would refine genetic counseling for patients and their families: even in somatic STK11-mutant STK11-AT, familial cancer history warrants expanded germline screening.

### 3.4 Treatment and future directions

Currently, no standardized adjuvant therapy exists for STK11-AT. Complete cytoreductive surgery (as performed here) remains the mainstay of treatment for recurrence, as it improves symptom control and potentially extends survival (4). Platinum-based chemotherapy is often used empirically, but its efficacy has not been validated in clinical trials.

Emerging preclinical data offer hope for targeted therapies:

#### 3.4.1 Immune checkpoint inhibitors

STK11 loss typically confers ICI resistance by reducing T-cell infiltration (15). However, Ahronian et al. (16) recently showed that TNG260 (a CoREST complex inhibitor) reverses this resistance by epigenetically reactivating immune-related genes (e.g., IFN-γ, CXCL10) in STK11-mutation tumors. This suggests combination therapy (TNG260 + anti-PD-1) could be effective for STK11-AT.

#### 3.4.2 mTOR inhibitors

STK11 loss activates mTOR signaling (1), making mTOR inhibitors (e.g., everolimus) a rational target. Preclinical studies in STK11-deficient lung cancer show promising activity (1), and extrapolation to STK11-AT is warranted.

### 3.5 Comparison with prior literature

The Recurrence interval, Tumor size (primary), Transition Mode, Ki-67 Index and PJS association were compared with previous published reports (**Table 2**). Therefore, the particularity of the patient’s condition in this case is highlighted. This case report can further enrich the sample bank of this disease, and provide some experience for workers in the same industry.

**Table 2:** Comparison of this case with previously reported cases.

Comparison dimension	The situation of this case	Literature reporting situation [(2) (4)]	Differences/particularities
Recurrence interval	9 months	Median overall survival of 17 months (19 months for PJS-related cases, 7 months for non-PJS-related cases)	Shorter than the median value, suggesting the possibility of a more aggressive sub-type
Tumor size (primary)	6x5cm(Left fallopian tube )	Average tumor size of 12.6 cm for PJS-related cases and 8.9 cm for non-PJS-related cases	Although smaller than the average reported in the literature, recurrence and progression were more rapid
Transition Mode	Peritoneal dissemination of para-aortic lymph node metastasis (3cm)	Presence of peritoneal dissemination and lymph node metastasis (predominantly pelvic)	The para-aortic lymph node metastasis was larger (3 cm), and the metastatic spread was extensive (from the pelvis → diaphragm → level of the renal vessels), demonstrating strong lymph node invasiveness
Ki-67 Index	70%	The general reference level was not explicitly mentioned	An extremely high proliferation index may be suggestive of rapid tumor progression (and/or recurrence)
PJS association	None (germline STK11 negative)	45% of patients exhibited PJS features or molecular alterations (17)	The clinical characteristics align with those of non-PJS-related STK11-altered tumors (STK11-AT), but a familial clustering of cancers is present (warranting further investigation)

## 4 Conclusion

This case refines the clinicopathological spectrum of STK11-AT by documenting ultra-rapid peritoneal dissemination, a high Ki-67 index, and familial cancer clustering in a somatic STK11-mutant patient. Key clinical recommendations include:

1. Definitive diagnosis: STK11 mutation testing is mandatory for adnexal tumors with ambiguous morphology (e.g., resembling FATWO or mesothelioma), as immunohistochemistry alone is insufficient.
2. This case suggests that a highly aggressive subtype may be present, and although validation in additional cases is needed, it is recommended that patients with STK11-AT and a high Ki-67 index require close surveillance (e.g., imaging every 3 to 4 months for 2 years after resection) to detect early recurrence.
3. Genetic counseling: Expanded germline panel testing is recommended for STK11-AT patients with a family history of cancer, even if STK11 germline testing is negative.
4. Therapy: Emerging targeted agents (CoREST inhibitors, mTOR inhibitors) warrant investigation in clinical trials for recurrent STK11-AT.
5. The main limitation of this study is the single-case design with limited follow-up time, which limits the generalizability of the findings. Further multi-institutional studies are needed to validate these findings and establish standardized diagnostic, surveillance, and therapeutic strategies for this aggressive neoplasm.

## Data Availability

The original contributions presented in the study are included in the article/supplementary material. Further inquiries can be directed to the corresponding authors.
