# Supplementary material for: Ultra-rapid peritoneal dissemination of a somatic STK11-mutant adnexal tumor: a case report with molecular correlation and literature review
Source: Front Oncol. 2026 Jul 24;16:1836973. doi: 10.3389/fonc.2026.1836973 (PMC13447140; doi:10.3389/fonc.2026.1836973)
Supplement: Supplementary file 2 [file DataSheet2.pdf]

Gene panel information

The 57 genes and regions included in this test were as follows: Ip190, AKT), ATRX, B2N, BAPJ, BCOR, BRAF,GCNDI, CDK4, CDK6.

CDKN2A,CDKN2B, CIC,CTNNBL, DDX3X, DICERL, BGFR, ERBB4, FGFRL, FGERL ex0n10-18 IID., FGFR2, FCFR3.

FOXL2, GNAIL, GNAQ, GNAS, H3F3A, HBF3B, HRAS, IDDI, IDI2, JAKL, JAK2, KIT.KRAS, MAP2KI, MAP2K2. NDM2.

MDNA, MET, MYODJ, NF1.NRAS PDCFRA, PDGPRB, PIK3CA, PTEN, RBd, SDIN SDB: 3B], SMARCA4, SMARCBI, STKIL, STK31, TERT (Promoter), 1P53.

All coding regions of 19 genes were tested, including: ATRX, B2N, BAP, CDKAZA, CDKN2B DOX3X. WDN2, NFL, PTEN, RBL, SDHA, SDHIB, SDHIE SDHID SMARCAL, SUAICRI, STKIL, STK31, 1P53:

Twenty-six genes were tested for CNV variants, including: ATRX, B2N, BAPL, CCNDH,CODK1, CDKG.CDKA2A, CIKNZB. CIC, EGFR, FCFRI, FGFRZ, FGFR3, JAKI, JAK2, MDN2, MDMA. WET, NPL, PTEN, RBJSHANCA4 SHARCBL, SIK11: SIK3i, TP53:

ITD was detected in 2 genes: BCOR and PGFRI.

Note: Due to the existence of 100% homologous sequences on the human genome, the kit cannot analyze mutations located in the following regions: SDIIC gene exon6 p. V155_*170 (chrl:161332176-161332258), PTEN gene exon9 p. E358 P387 (chr-10:89725089-)
89725176).

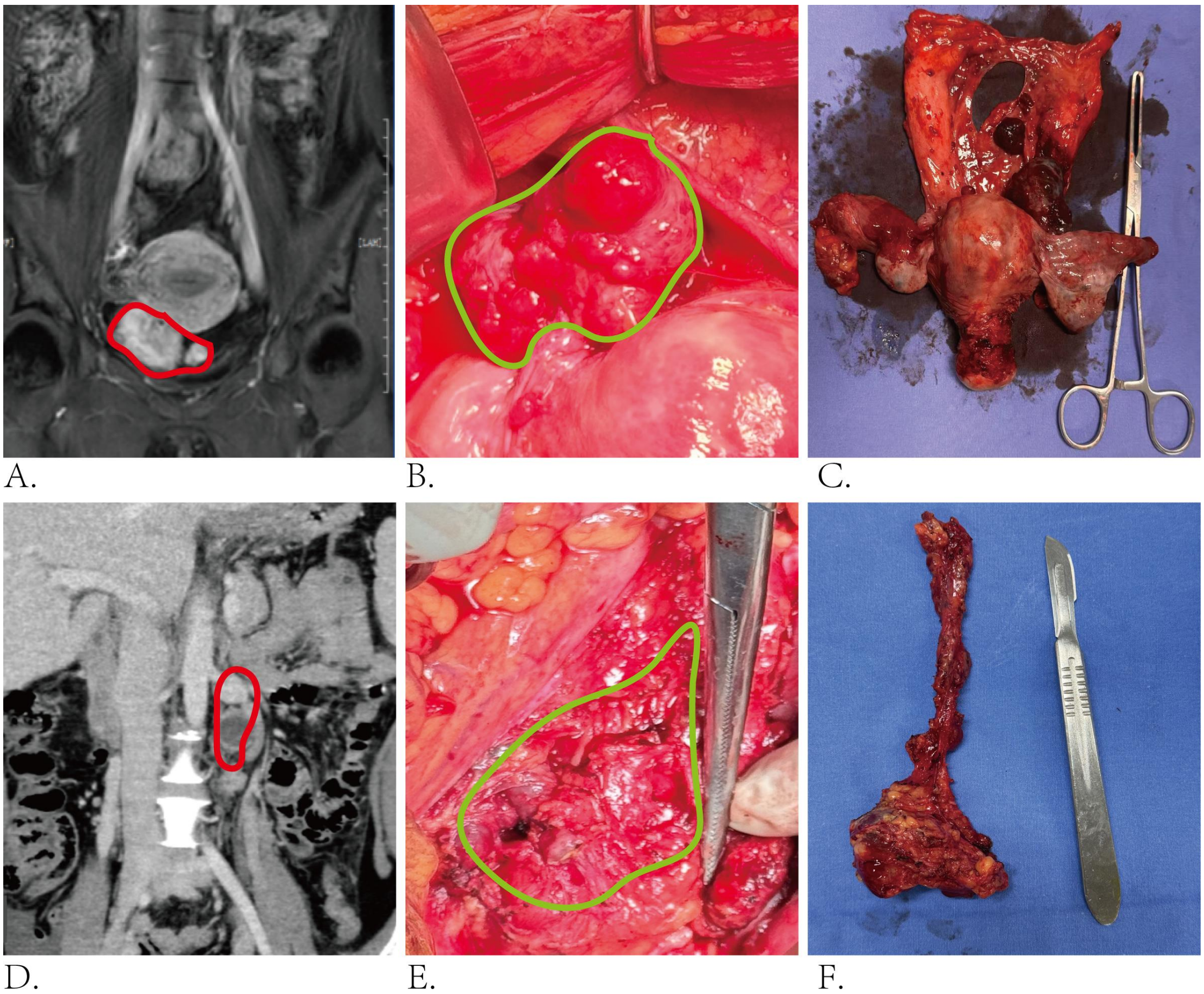

Figure 3.Preoperative imaging, intraoperative and postoperative gross pathological findings of patients with pelvic masses (A-C) and para-aortic enlarged lymph nodes (D-F).
